# ERRATUM

**DOI:** 10.1590/1806-9282.20230454ERRATUM

**Published:** 2024-03-04

In the manuscript “miR604A>G gene polymorphism is associated with recurrent pregnancy loss in Turkish women”, https://doi.org/10.1590/1806-9282.20230454, published in the Rev Assoc Med Bras. 2023;69(9):e20230454, on page 1:

**Where it reads:**

The study was approved by the Clinical Research Ethics Committee of Ondokuz Mayıs University Faculty of Medicine on May 31, 2023 (OMU KAEK 2023/178).

**It should read:**

The study was approved by the Clinical Research Ethics Committee of Ondokuz Mayıs University Faculty of Medicine on November 14, 2019 (OMU KAEK 2019/744).

